# Deoxycholic Acid Upregulates Serum Golgi Protein 73 through Activating NF-κB Pathway and Destroying Golgi Structure in Liver Disease

**DOI:** 10.3390/biom11020205

**Published:** 2021-02-02

**Authors:** Danli Yang, Mingjie Yao, Ying Yan, Yanna Liu, Xiajie Wen, Xiangmei Chen, Fengmin Lu

**Affiliations:** 1 Department of Microbiology and Infectious Disease Center, School of Basic Medical Science, Peking University Health Science Center, Beijing 100191, China; ydl0310@pku.edu.cn (D.Y.); yanying@bjmu.edu.cn (Y.Y.); 2016395001@bjmu.edu.cn (Y.L.); wenxiajie@pku.edu.cn (X.W.); 2 Department of Anatomy and Embryology, School of Basic Medical Sciences, Peking University Health Science Center, Beijing 100191, China; 3 Hepatology Institute, Peking University People’s Hospital, Beijing 100044, China; 4 Center for Precision Medicine, Academy of Medical Sciences, Zhengzhou University, Henan 450052, China

**Keywords:** liver disease, bile acids, Golgi protein 73, deoxycholic acid, nuclear factor-kappa B

## Abstract

Golgi protein 73 (GP73) is upregulated in a variety of liver diseases, yet the detailed mechanism is poorly characterized. We analyzed GP73 in a retrospective cohort including 4211 patients with chronic liver disease (CLD) or hepatocellular carcinoma (HCC). The effect of deoxycholic acid (DCA) and nuclear factor-kappa B (NF-κB) on expression and release of GP73 in Huh-7 and SMMC7721 cells were studied. A mouse study was used to confirm our findings in vivo. A positive correlation was found between serum GP73 and total bile acid (TBA) in cirrhotic patients (*r* = 0.540, *p* < 0.001), higher than that in non-cirrhotic CLD (*r* = 0.318, *p* < 0.001) and HCC (*r* = 0.353, *p* < 0.001) patients. In Huh-7 and SMMC7721 cells, DCA upregulated the expression and release of GP73 in a dose- and time-dependent manner. After overexpressing NF-κB p65, the promoter activity, GP73 messenger RNA (mRNA) level, and supernatant GP73 level were increased. The promotion effect of DCA on GP73 release was attenuated after inhibiting the NF-κB pathway. Mutating the binding sites of NF-κB in the sequence of the GP73 promoter led to a declined promoting effect of DCA on GP73. The upregulation role of DCA in GP73 expression through the NF-κB pathway was confirmed in vivo. In addition, exposure to DCA caused disassembly of Golgi apparatus. In summary, DCA upregulates the expression and release of GP73 via activating the NF-κB pathway and destroying the Golgi structure.

## 1. Introduction

Chronic liver disease (CLD) is one of the significant public health problems and accounts for approximately 1.5 million deaths per year globally [1,2,3]. CLD may eventually progress to end-stage liver disease and even hepatocellular carcinoma (HCC), which is estimated to be the fourth most common cause of cancer-related death worldwide [4,5]. It has been reported that fibrosis and even early-stage cirrhosis could be reversed by prompt clinical intervention [6]. Thus, developing potential biomarkers for staging and monitoring the disease progression and regression and uncovering the underlying mechanism is of great importance.

Golgi protein 73 (GP73) is a transmembrane protein of the Golgi apparatus with a relative molecular weight of 73 kD, which was first reported by Kladney et al. in 2000 [7]. GP73 is mainly expressed in human bile duct epithelial cells while it is rarely expressed in hepatocytes [7,8]. However, GP73 was shown to be upregulated in a variety of liver diseases, including alcohol-related liver disease, viral hepatitis, and HCC [9,10]. Although accumulating studies have suggested that serum GP73 might be a reliable and accurate biomarker for staging liver fibrosis and cirrhosis in CLD patients with different etiologies [11,12], the molecular mechanism relevant to the aberrant elevation of serum GP73 in liver diseases remains largely unknown.

Bile acids are the main component of bile, accounting for about 50% to 70% of the total bile. As important signaling molecules, bile acids serve as the natural ligands of diverse nuclear receptors and membrane receptors, and they are involved in the metabolism regulation of lipids, glucose, energy, and a variety of drugs [13]. Bile acids can also affect the Golgi apparatus and impair protein processing, transport, and secretion [14,15]. Bile acids such as glycine deoxycholic acid (DCA) and taurocholic acid are associated with liver cirrhosis and could be used as potential biomarkers for liver cirrhosis [16]. Recently, a study found that stimulating esophageal cancer cells with DCA could destroy and break down the structure of the Golgi apparatus, resulting in increased secretion of GP73 [15]. Additionally, our previous study observed that level of serum GP73 was independently positively correlated with total bile acid (TBA) in HCC patients [17]. However, it is unknown whether DCA plays a role in upregulating the level of serum GP73 in liver disease.

In this study, we discovered that DCA treatment can upregulate GP73 expression and be released both in vitro and in vivo. Activation of nuclear factor-kappa B (NF-κB) by DCA might be the potential mechanistic link. In addition, we demonstrated that the DCA-caused Golgi structure disassembly may be another mechanism for promoting GP73 release. Therefore, our research reveals the mechanism of serum GP73 elevation in liver diseases and hints that modulating of bile acids or the NF-κB pathway may uncover new therapeutic strategies for liver disease.

## 2. Materials and Methods

### 2.1. Patients

A total of 4211 CLD and HCC patients admitted to the Fifth Medical Center of the Chinese PLA General Hospital were retrospectively enrolled between January 2010 and November 2017. The inclusion and exclusion criteria were described in our previous study [11]. The METAVIR scoring system was used to evaluate the fibrosis stage, which was graded from F0 to F4. Diagnosis of cirrhosis was based on liver biopsy and/or clinical findings including imaging (CT or MRI), esophagogastroduodenoscopy examinations, and laboratory tests. The study was approved by the Ethics Committee of Fifth Medical Center of Chinese PLA General Hospital (approval code: No. 2020-0657).

### 2.2. Animals

Male C57BL/6 mice (7 weeks old) were purchased from Department of Laboratory Animal Science of Peking University Health Science Center (Beijing, China). Mice were randomly divided into two groups with 11 mice in each group and were intragastrically administered 250 μL of DCA (8 mg/mL) or phosphate-buffered saline (PBS) for 21 days. Mice were sacrificed after overnight fasting. Blood and liver tissues were collected. All operations on animals followed internationally recognized guidelines on laboratory animal use.

### 2.3. Cell Lines

Huh-7 was purchased from the American Type Culture Collection (Manassas, VA, USA) and cultured in Dulbecco’s modified Eagle’s medium supplemented with 10% fetal bovine serum (Gibco, Life Technologies, Carlsbad, CA, USA). SMMC7721 was purchased from Cell Resources Center of Peking Union Medical College (Beijing, China) and cultured in Roswell Park Memorial Institute (RPMI)-1640 supplemented with 10% fetal bovine serum (Gibco, Life Technologies, Carlsbad, CA, USA). Short tandem repeat typing, hepatic gene expression, and specific gene mutations were detected to prove the origin of SMMC7721.

### 2.4. Quantification of GP73 in Cell Culture Supernatant

The concentration of GP73 in the cell culture supernatant was quantified using a commercially available GP73 quantitative determination kit (up-conversion luminescence method, Beijing Hotgen Biotech Co., Ltd., Beijing, China) in the MQ60 series automatic chemiluminescence immunoassay analyzer (Beijing Hotgen Biotech Co., Ltd., Beijing, China).

### 2.5. Quantification of Serum GP73, TBA, Alanine Transaminase (ALT), and Aspartate Transaminase (AST)

Mouse serum GP73 was analyzed using the ELISA kit following the manufacturer’s instructions (Cloud-Clone, Wuhan, China). Mouse serum TBA, ALT, and AST were quantified by the Laboratory Medicine Department of Peking University Third Hospital (Beijing, China). Mouse serum was diluted fivefold with PBS.

### 2.6. RNA Extraction and Quantitative Reverse-Transcriptase PCR (qRT-PCR)

RNA extraction and qRT-PCR were performed as described previously [18]. The primers used for qRT-PCR are listed in Table S1 (Supplementary Materials).

### 2.7. Western Blot (WB) Analysis

WB analysis was performed as previously described [18]. First, the lysed cell supernatant was run on SDS-PAGE and then blotted with antibodies. Antibodies used in WB were as follows: anti-GP73 (Abcam, Cambridge, UK), anti-P65 (Cell Signaling Technology, Boston, MA, USA), anti-phosphorylated P65 (ImmunoWay Biotechnology Company, Plano, TX, USA), anti-IκBα, anti-phosphorylated IκBα, anti-IKKα/β (Santa Cruz Biotechnology, CA, USA), anti-GAPDH, and anti-β-actin (Abgent, San Diego, CA, USA).

### 2.8. Luciferase Reporter Assay

Dual-luciferase reporter assays were carried out as described previously [19]. The conserved region of the human GP73 promoter was amplified using PrimeSTAR HS DNA Polymerase (Takara, Dalian, China) and inserted into pGL3-Basic plasmid to construct wildtype and binding-site-mutated GP73 promoter luciferase reporter vectors. All primers used are shown in Table S1 (Supplementary Materials).

### 2.9. Cytoimmunofluorescence

Immunofluorescence assays were carried out as described previously [18]. Cells were fixed in 4% paraformaldehyde at room temperature for 20 min, permeabilized with 0.3% Triton X-100 for 10 min, and subsequently blocked with PBS containing 5% goat serum. Cells were incubated overnight with primary antibody P65 (Cell Signaling Technology, Boston, USA), GP73 (Abcam, Cambridge, UK), or GM130 (Santa Cruz Biotechnology, CA, USA) at 4 °C, followed by secondary antibodies tetramethylrhodamine (TRITC)-conjugated goat anti-mouse immunoglobulin G (IgG) (Origene, MD, USA) or fluorescein isothiocyanate (FITC)-conjugated goat anti-rabbit IgG (Origene, MD, USA) for 1 h at room temperature. Lastly, the samples were stained with Hoechst (10 μg/mL) at room temperature for 10 min and photographed with a confocal laser microscope (Leica TCS-SP8, Heidelberg, Germany).

### 2.10. Chromatin Immunoprecipitation (ChIP) Assay

The ChIP assay was performed as described previously [18]. Briefly, Huh-7 and SMMC7721 cells were transfected with the NF-κB p65 expression plasmid. The lysates were incubated with rabbit anti-P65 (Cell Signaling Technology, Boston, USA) or immunoglobulin G from rabbit serum (Cell Signaling Technology, Boston, USA). The ChIP assay of mouse liver tissues was performed according to the manufacturer’s instructions (Cell Signaling Technology, Boston, USA).

### 2.11. Statistical Analysis

All statistical analyses were performed using the SPSS 24.0 software (IBM Corp., Armank, NY, USA) or GraphPad Prism7 (Graphpad, La Jolla, CA, USA). Data were presented as median (interquartile range) or mean ± standard error of the mean (SEM). Fisher’s exact probability test was used to compare categorical variables. The values between two groups were analyzed using a two-tailed Student’s *t-*test. A one-way ANOVA test was used for comparison of multiple groups. A Spearman test was used to assess the correlation between parameters. A *p-*value < 0.05 was considered significant.

## 3. Results

### 3.1. Serum GP73 and TBA Are Significantly Increased in Patients with CLD or HCC

**Figure 1:**
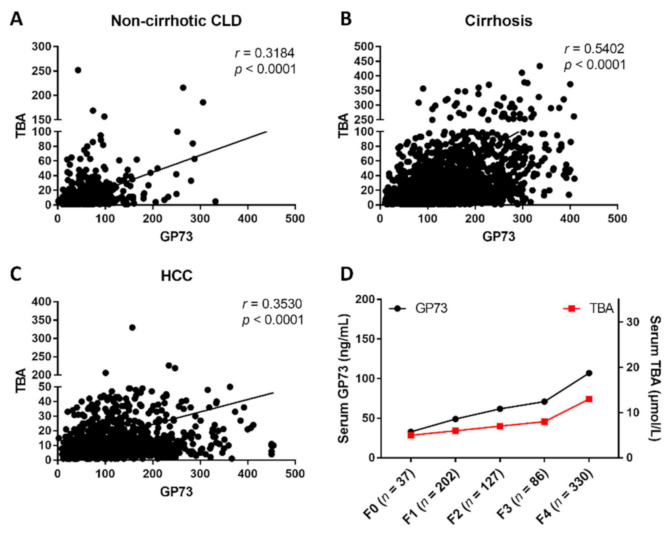
The correlation between serum Golgi protein 73 (GP73) and total bile acids (TBA) in patients with chronic liver disease (CLD) and different fibrosis stages. The correlation of serum GP73 and TBA in patients with non-cirrhotic CLD (**A**), cirrhosis (**B**), and HCC (**C**). (**D**) Serum levels of GP73 and TBA in CLD patients with different fibrosis stages. Statistical analysis was performed using GraphPad Prism7.

To investigate the relationship between the serum GP73 and TBA levels in liver disease, the serum GP73 and TBA levels were analyzed in a total of 4211 patients, including 985 non-cirrhotic CLD patients, 2141 liver cirrhosis patients, and 1085 HCC patients (Figure S1, Supplementary Materials). The demographic and clinical characteristics of these patients are summarized in Table 1. The results showed that CLD patients with cirrhosis had the highest serum GP73 level (127.90 ng/mL, 82.56 ng/mL–192.40 ng/mL) compared to patients with HCC (100.50 ng/mL, 60.62 ng/mL–161.50 ng/mL, *p* < 0.001) and non-cirrhotic CLD (46.42 ng/mL, 30.60 ng/mL–66.90 ng/mL, *p* < 0.001). In addition, both serum GP73 (117.34 ng/mL) and TBA (30.49 μmol/L) levels were significantly higher in CLD patients than the upper limit of normal GP73 (45 ng/mL, *p* < 0.001) and TBA (10 μmol/L, *p* < 0.001). A significant positive correlation was found between serum GP73 and TBA (*r* = 0.540, *p* < 0.001) in patients with cirrhosis, higher than that in non-cirrhotic CLD (*r* = 0.318, *p* < 0.001) and HCC patients (*r* = 0.353, *p* < 0.001) (Figure 1A–C). In the gradual progress from liver fibrosis to cirrhosis, both GP73 and TBA exhibited a simultaneous upward trend (Figure 1D).

### 3.2. DCA Upregulates the Expression of Endogenous GP73

**Figure 2:**
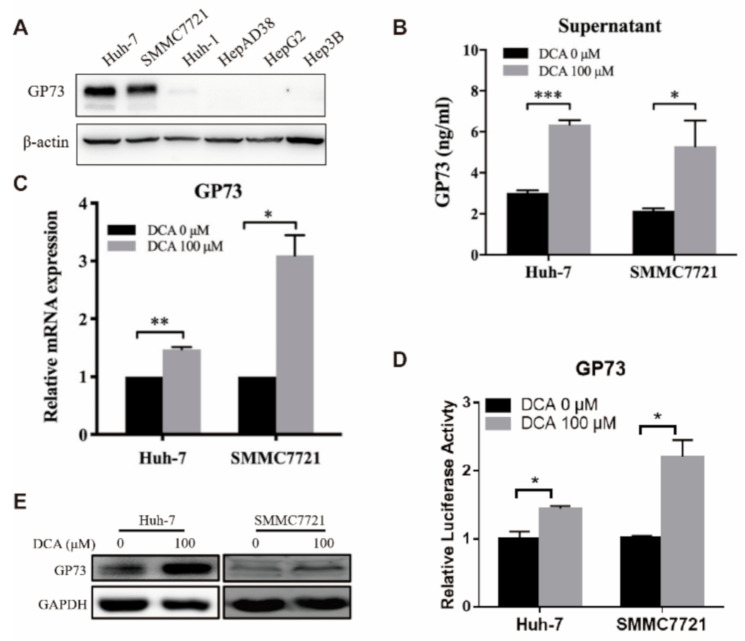
Deoxycholic acid (DCA) upregulates the expression of endogenous Golgi protein 73 (GP73). (**A**) The basal expressions of GP73 in Huh-7, SMMC7721, Huh-1, HepAD38, HepG2, and Hep3B cell lines were detected by Western blot (WB). Huh-7 and SMMC7721 cells were treated with 100 μM DCA for 72 h. (**B**) The cell supernatant level of GP73 was determined by ELISA. (**C**) Huh-7 and SMMC7721 cells were treated with or without 100 μM DCA for 72 h. The messenger RNA (mRNA) levels of GP73 were determined by qRT-PCR. (**D**) Huh-7 and SMMC7721 cells were transfected with PGL3 vector containing promoter of GP73 (PGL3-GP73) and treated with or without 100 μM DCA. Samples were harvested 48 h after treatment, and luciferase activities were measured. (**E**) Huh-7 and SMMC7721 cells were treated with or without 100 μM DCA for 72 h. The protein levels of GP73 were determined by WB. Data in B, C, and D are expressed as the mean ± standard error of the mean (SEM) and represent three independent experiments (* *p* < 0.05; ** *p* < 0.01; *** *p* < 0.001; ns: not significant). Statistical analysis was performed using GraphPad Prism7.

To investigate the regulation of bile acids with respect to GP73 level, we detected the endogenous GP73 expression and GP73 release in HCC cell lines after DCA treatment. First, the expression level of GP73 was detected in six human hepatoma cell lines by WB assay, and the results showed that two cell lines (Huh7 and SMMC7721) had the highest GP73 expression level and were selected for further studies (Figure 2A). As compared with that of DCA-untreated cells, a significantly higher level of GP73 in the supernatant of DCA-treated (100 μM) cells was observed, which suggested that DCA can promote the release of GP73 (Figure 2B), and this promotion effect was in a time- and dose-dependent manner (Figure S2A–D, Supplementary Materials).

Next, we explored the regulation of DCA on the transcription level of GP73. The messenger RNA (mRNA) expression levels of GP73 in Huh-7 and SMMC7721 cells both increased after treated with DCA (Figure 2C). Although the increase in RNA level was also in a dose- and time-dependent manner, unlike the supernatant levels that increased significantly at 48 h, the RNA levels of GP73 only increased significantly at 72 h after DCA treatment (Figure S3A–D, Supplementary Materials). The highest level of endogenous GP73 expression in Huh-7 was observed when treated with 200 μM of DCA. However, this concentration could lead to obvious cell death. Thus, an optimal concentration of 100 μM was chosen for the remaining experiments in our study. In line with the upregulation of GP73 at the mRNA level, the transcriptional activity of the GP73 promoter (Figure 2D) and its protein level also increased in Huh-7 and SMMC7721 cells exposed to DCA (Figure 2E). All the results above proved that DCA could upregulate endogenous GP73 expression in Huh-7 and SMMC7721 cells.

### 3.3. DCA Upregulates the Expression of Endogenous GP73 through NF-κB Pathway Activation

**Figure 3:**
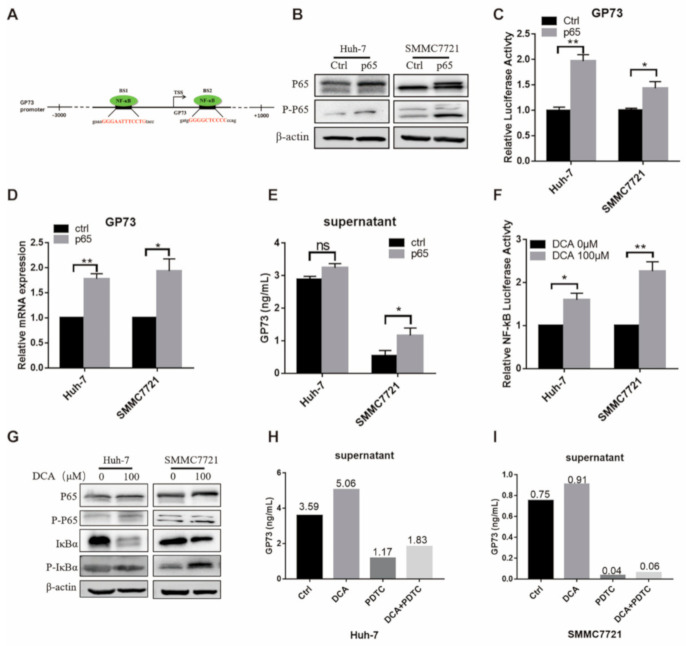
Deoxycholic acid (DCA) upregulates the expression of endogenous Golgi protein 73 (GP73) through activation of the NF-κB pathway. (**A**) Schematic diagram of NF-κB binding sites on GP73 promoter. (**B**–**D**) Huh-7 and SMMC7721 cells were transfected with PCMV-p65 or control PCMV vectors for 72 h. Samples were collected and a Western blot assay of NF-κB P65 and P-P65 (**B**), detection of cell supernatant level of GP73 by ELISA (**C**), and qRT-PCR assay of GP73 mRNA level (**D**) were performed. (**E**) Huh-7 and SMMC7721 cells were transfected with PGL3-GP73 together with control vector PCMV or PCMV-p65. Luciferase activities were measured 48 h later. (**F**) Huh-7 and SMMC7721 cells were transfected with an NF-κB-dependent firefly luciferase construct and treated with 100 μM DCA. Samples were harvested 48 h after treatment, and luciferase activities were measured. (**G**) Huh-7 and SMMC7721 cells were treated with 100 μM DCA for 72 h. NF-κB pathway proteins were determined by Western blot assay. Cell supernatant levels of GP73 in Huh-7 (**H**) and SMMC7721 (**I**) cells exposed to 0.1% dimethyl sulfoxide (DMSO, ctrl) (Ctrl), 100 μM DCA, 50 μM pyrrolidine dithiocarbamate (PDTC), or 100 μM DCA plus 50 μM PDTC were detected by ELISA. Data in (**C**–**F**) are expressed as the mean ± SEM and represent three independent experiments (* *p* < 0.05; ** *p* < 0.01; *** *p* < 0.001; ns: not significant). Statistical analysis was performed using GraphPad Prism7.

According to the JASPAR database (http://jaspar.genereg.net), there are two potential binding sites of NF-κB on the GP73 promoter (Figure 3A). To investigate whether the NF-κB pathway is involved in the DCA-induced GP73 expression, we firstly explored if activation of the NF-κB pathway affects the expression and release of GP73. After overexpressing NF-κB p65 in Huh-7 and SMMC7721 cells, both total and phosphorylated NF-κB P65 were increased (Figure 3B). Simultaneously, the promoter activity, the mRNA level of GP73, and the content of GP73 in supernatant were all increased in the p65 overexpression group (Figure 3C–E). Next, we discovered the effect of DCA on the activity of NF-κB pathway by using am NF-κB-dependent firefly luciferase construct. The luciferase assay showed that DCA treatment increased the NF-κB activities in both Huh-7 and SMMC7721 cells (Figure 3F). Then the WB assay was performed to detect the protein level of total NF-κB P65, phosphorylated NF-κB P65, inhibitory subunit of NF-κB Alpha (IκBα), and phosphorylated IκBα. The results showed that, when Huh-7 and SMMC7721 cells were exposed to DCA, the total NF-κB P65, p-NF-κB P65, and P-IκBα were increased, while total IκBα was decreased (Figure 3G), suggesting activation of the NF-κB pathway in response to DCA treatment. Taken together, the above results demonstrated that DCA treatment could activate the NF-κB pathway in Huh-7 and SMMC7721 cells. After pyrrolidine dithiocarbamate (PDTC), an effective inhibitor of the NF-κB pathway, was added to the culture medium of Huh-7 and SMMC7721 cells, the promotion effect of DCA on GP73 release was attenuated (Figure 3H,I). Taken together, these results supported the hypothesis that DCA upregulates the expression of endogenous GP73 and promotes the release of GP73 through activation of the NF-κB pathway.

### 3.4. NF-κB P65 Directly Binds to the Promoter of GP73 to Upregulate Its Expression

**Figure 4:**
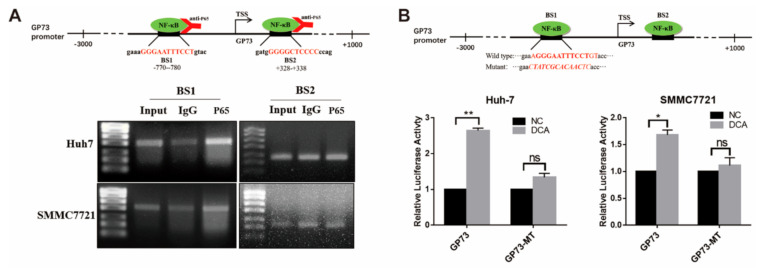
NF-κB P65 directly binds to the promoter of Golgi protein 73 (GP73). (**A**) Schematic diagram of chromatin immunoprecipitation (ChIP) PCR (upper). The products of ChIP-PCR in the input, immunoglobulin G (IgG), and ChIP groups were analyzed using agarose gel electrophoresis (lower). (**B**) Schematic diagram of NF-κB P65 binding site 1 mutant (upper). Huh-7 and SMMC7721 cells were transfected with PGL3-GP73 or GP73-MT and treated with or without 100 μM deoxycholic acid (DCA). Samples were harvested 48 h after treatment, and luciferase activities were measured (lower). Data in B are expressed as the mean ± SEM and represent three independent experiments (* *p* < 0.05; ** *p* < 0.001; ns: not significant). Statistical analysis was performed using GraphPad Prism7.

Next, how NF-κB regulates the transcription of GP73 was explored. Bioinformatics analysis identified two potential NF-κB binding sites (BS1: −780/−770; BS2: +328/+338) in the promoter sequence of GP73 (Figure 4A). The chromatin immunoprecipitation (ChIP) PCR analysis demonstrated that NF-κB P65 directly interacted with the −780/−770 site (BS1) of the GP73 promoter. To verify if such binding affects the transcription activity of the GP73 promoter, a GP73 promoter BS1 mutant vector (GP73-MT) was constructed (Figure 4B). The results of luciferase activity assay revealed that, when the binding sites were mutated, the promoting effect of DCA on GP73 promoter activity declined from 1.33- to 2.65-fold in Huh-7 cells and from 1.11- to 1.68-fold in SMMC7721 cells (Figure 4B), which indicated that NF-κB P65 lost its interaction with the GP73 promoter. The above data elucidated that upregulation of NF-κB P65 promoted the transactivation of GP73 via directly binding to the GP73 promoter, and DCA upregulated GP73 expression through NF-κB pathway activation.

GP73 is located on the *cis*-Golgi membrane. When GP73 reaches the *trans*-Golgi, it is cleaved by preprotein converting enzymes (PCs) at the 55th amino acid to form a secreted GP73 protein. In vitro studies showed that Furin can cleave at the R52VRR55 site of GP73 protein [20]. We explored whether the NF-κB activation can upregulate Furin expression, thereby promoting the release of GP73 in Huh-7 and SMMC7721 cells. It was revealed that both the promoter activity and the mRNA expression levels of Furin were elevated by NF-κB p65 overexpression in Huh-7 and SMMC7721 cells (Figure S4A,B, Supplementary Materials). However, increased Furin protein level after DCA treatment was only seen in SMMC7721 cells (Figure S4C, Supplementary Materials). Unexpectedly, instead of promoting the secretion of GP73, ectopic expression of Furin in the Huh-7 cell line reduced the secretion of GP73. Compared with the overexpression of GP73 alone, the secretion of GP73 also decreased when co-transfected with Furin-expressing plasmid DNAs (Figure S4D, Supplementary Materials).

### 3.5. DCA Upregulates NF-κB-Mediated GP73 Expression In Vivo

**Figure 5:**
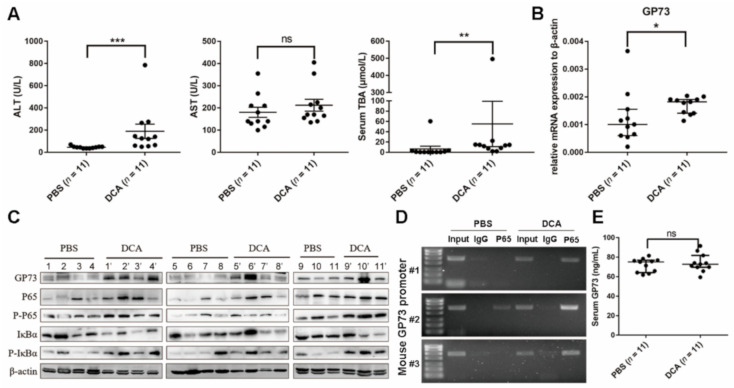
Deoxycholic acid (DCA) upregulating Golgi protein (GP73) expression through NF-κB pathway in mice. (**A**) Male C57BL/6 mice were sacrificed at 21 days after being administered phosphate-buffered saline (PBS) or DCA. ALT, AST, and TBA in serum were analyzed. (**B**) qRT-PCR analysis was carried out to determine GP73 expression level in liver tissues of C57BL/6 mice administered with PBS or DCA for 21 days. (**C**) GP73 and NF-κB pathway proteins of C57BL/6 mice administered PBS or DCA for 21 days were determined by WB (11 mice for each group). (**D**) ChIP-PCR assay to determine the binding of NF-kB P65 on GP73 promoter in mouse liver tissues. Three liver tissues from PBS and DCA administration group were randomly selected. The products of ChIP-PCR in the input, IgG, and ChIP groups were analyzed using agarose gel electrophoresis. (**E**) Serum GP73 levels were determined by ELISA. Data in (**A**,**B**,**D**) are expressed as the mean ± SEM (* *p* < 0.05; ** *p* < 0.01; *** *p* < 0.001; ns: not significant). Statistical analysis was performed using GraphPad Prism7.

Compared to mice administered PBS, the serum ALT and TBA levels of mice administered DCA were significantly increased (*p* < 0.0001) (Figure 5A), while AST level showed no statistical difference between two groups (*p* > 0.05), which implied the destructive effects of excess bile acids on liver cell membranes. qRT-PCR analysis showed that DCA-administered mice exhibited a lower cholesterol 7α-hydroxylase (Cyp7a1) mRNA level (*p* < 0.0001), which is the rate-limiting enzyme for bile acid synthesis. Yet, the RNA expression levels of sodium taurocholate cotransporting polypeptide (NTCP), bile salt export pump (BSEP), and organic anion transporting polypeptide (OATP) 1α1, 1β2, and 2β1, which are important transport proteins in bile acid enterohepatic circulation, showed no changes (*p* > 0.05; Figure S5, Supplementary Materials). As shown in Figure 5B,C, the mRNA expression and protein levels of GP73 both increased in mice exposed to DCA. WB analysis revealed the elevated protein level of NF-κB P65, P-P65, and P-IκBα and decreased total IκBα protein level in DCA-administered mice (Figure 5C), suggesting that DCA administration activates the NF-κB pathway in vivo. The ChIP-PCR assay revealed NF-κB P65 to be elevated by DCA administration bound to the promoter of GP73 (Figure 5D). These results collectively demonstrated that DCA could activate the NF-κB pathway to upregulate GP73 expression in vivo. Unexpectedly, there was no statistical difference in serum GP73 levels between the two groups (Figure 5E).

### 3.6. DCA May Promote GP73 Release by Causing Golgi Structure Disassembly

**Figure 6:**
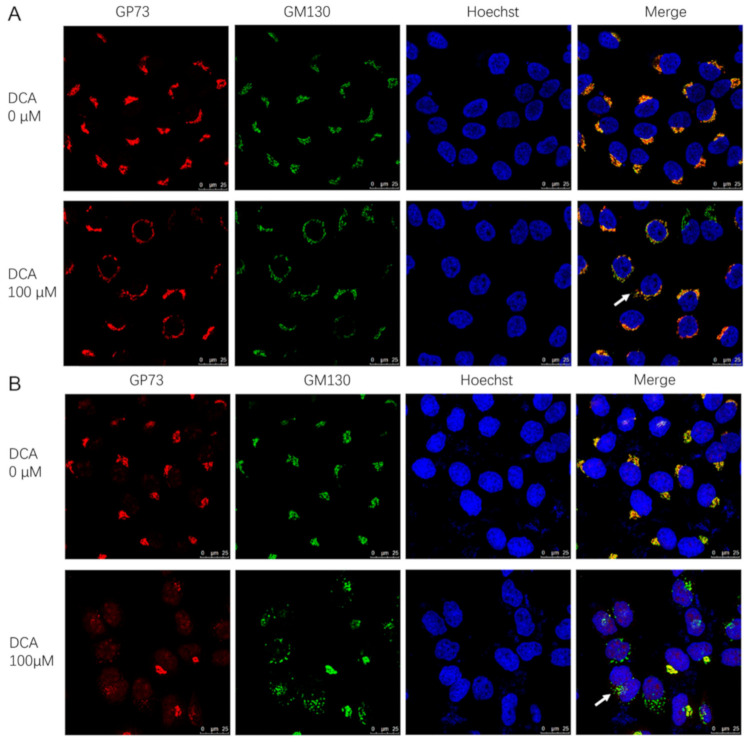
Deoxycholic acid (DCA) converts the Golgi into ministacks in Huh-7 and SMMC7721 cells. Huh-7 cells (**A**) and SMMC7721 cells (**B**) were treated with 100 μM DCA for 72 h and co-stained for GP73 (red) and *cis*-Golgi marker GM130 (green); nuclei were identified with Hoechst (blue). Arrows denote the ministacks.

In addition to upregulating the gene expression of GP73, the sabotage effect of DCA on biological membranes may be another mechanism for GP73 release promotion. To determine whether this was the case, both *cis*-Golgi network marker (GM130) and GP73 staining were visualized in Huh-7 and SMMC7721 cell lines. In DCA-untreated cells, GM130 and GP73 colocalized as a single perinuclear ribbon structure, indicating a normal intact Golgi structure and GP73 localization. On the contrary, exposure to DCA for 72 h caused the disassembly of Golgi into multiple rings or ministacks (Figure 6A,B), which indicated the transport capability enhancement of GP73 to the membrane. In fact, the disassembly of Golgi could be seen as early as 6 h after DCA treatment in Huh-7 cells (Figure S6, Supplementary Materials). These results revealed that, in response to DCA, the intact Golgi structure was scattered, which consequently led to the release of GP73. 

## 4. Discussion

GP73 is a type II transmembrane glycoprotein expressed in the epithelial lineage tissues and plays important roles in liver growth and development [21,22]. In patients with liver disease, a significant elevation of serum GP73 is observed [9], making it a potential biomarker for staging liver disease progression [23]. However, the underlying mechanism remains poorly characterized. In the present study, we illustrate a novel mechanism via which bile acids upregulate the expression and release of GP73 in an NF-κB-dependent manner, which may help to better understand the roles of GP73 in liver disease.

Bile acids are synthesized, excreted, and reabsorbed in the liver, and they are responsible for solubilization of the lipolysis products, digestion, and lipid absorption [24]. It has been reported that the dysregulation of bile acid levels and speciation resulted in cytotoxicity, inflammation, and fibrosis during liver injury [25]. In a clinical setting, TBA is the only important indicator that can simultaneously reflect the capacities of liver secretion, synthesis, and metabolism, as well as the status of liver cell injury. Since the uptake and metabolic function of bile acids by liver cells are damaged in CLD patients, the peripheral blood TBA is elevated. In the present study, a significant increase in serum GP73 and TBA was observed in our CLD cohort compared with the normal population. Additionally, significant positive correlations between serum GP73 and TBA levels were found in patients with non-cirrhotic CLD, cirrhosis, and HCC, and both parameters increased as liver fibrosis stage progressed. These results suggested that bile acids correlate with GP73 in the progression of liver disease.

DCA, mainly produced in the cecum and proximal colon, is one of the naturally occurring secondary bile acids generated by anaerobic bacteria degrading bound bile acids through glycocholic acid hydrolase and 7α dehydroxylase [26]. Its potential procarcinogenic and proinflammatory actions lead to its uniqueness among bile acids [27,28,29]. It has been reported that DCA promotes the development of Barrett’s esophagus and esophageal disease [15,30,31]. In addition, DCA has been reported to be strongly associated with the occurrence of HCC [32]. In the present study, the role of DCA in the expression and release of GP73 was studied in detail. We found that DCA not only upregulated the release of GP73, but also the expression of GP73 in a dose- and time-dependent manner. These results suggested that DCA may be widely involved in the progression of liver disease, which may be one of the reasons for the increased release of GP73. In addition, bile acids have strong interfacial activity, which can reduce the surface tension between oil and water and help the digestion and absorption of fats and lipids in food [33]. They can destroy the lipid components of the cell membrane and cause oxidative damage [34]. In Huh-7 and SMMC7721 cells, DCA was found to partially destroy the structure of the Golgi apparatus, which may be another mechanism for DCA-promoted GP73 release. Future study is needed to determine the Golgi structure changes following in vivo DCA administration in mice using electron microscopy. Previous studies provided some insights into the regulation mechanisms of GP73 expression via activation of the mammalian target of rapamycin (mTOR) pathway and inflammation-related factors such as Interleukin-6 (IL-6) in liver disease [35,36,37,38]. The results of the present study revealed that NF-κB, serving as a transcription promoter, could activate GP73 expression. A previous study reported that, in esophageal epithelial cells, DCA can induce NF-κB DNA-binding activity in a dose- and time-dependent manner [39]. Consistently, we confirmed that DCA activates the NF-κB pathway both in vivo and in vitro. In HCC cell lines, DCA could activate the NF-κB pathway as phosphorylated P65 increased and total IκBα decreased. However, a dose- and time-dependent effect of DCA on the NF-κB pathway was not detected in our study. The activated NF-κB pathway not only increases the mRNA and protein expression of GP73 but also the release of GP73, building the link between DCA and GP73. In DCA-gavage mice, an increase in GP73 mRNA and protein levels in liver tissue and activation of the NF-κB pathway were also observed. Mechanistically, we demonstrated that NF-κB P65 can directly bind to the promoter of GP73. However, no statistical difference in serum GP73 levels was seen between PBS- and DCA-gavage mice. Considering that (i) GP73 has a stronger correlation with AST (*r* = 0.530, *p* < 0.001) than ALT (*r* = 0.231, *p* < 0.001; data not shown) in this cohort, and (ii) no significant difference of serum AST level was found between two groups, this indifference in serum GP73 levels may be as a result of the slight tissue damage, which is not enough to cause GP73 to be effectively cut and released. Meanwhile, we quantitatively analyzed mRNA expressions of bile acid synthesis- and transport-related proteins in DCA-gavage mice compared to PBS-gavage mice. It was revealed that, except for Cyp7a1, the rate-limiting enzyme for bile acid synthesis, the expression levels of other bile acid transport-related proteins showed no statistical difference between the two groups, suggesting a normal enterohepatic circulation remains in DCA-gavage mice. The decline in Cyp7a1 in DCA-gavage mice may be compensatory downregulation in response to exogenous DCA.

As Furin can cleave GP73 at the 55th amino acid at the N-terminus [20], the expression change of Furin was detected in our research. Although the promoter activity and mRNA level were upregulated when the NF-κB pathway was activated in both Huh-7 and SMMC7721 cells, the protein level of Furin was only slightly increased in SMMC7721 cells, but not in Huh-7 cells. A previous report showed that, in HeLa cells, Furin overexpression alone failed to increase GP73 release, which is consistent with our results, suggesting that the upregulation of Furin in liver cancer, if it occurs, may not be the only potential mechanism for the increased GP73 serum levels [20]. Therefore, whether the protein level of Furin responds to NF-κB pathway activation may not directly relate to the increased secretion of GP73.

In summary, this study indicated how DCA regulates the expression and release of GP73 in Huh-7 and SMMC7721 cell lines via activating the NF-κB pathway and destroying the Golgi structure. This study helps to explain the molecular mechanism of elevated serum GP73 in patients with CLD and HCC, and it provides experimental evidence to support GP73 as a serological marker for diagnosis of liver disease. Furthermore, the present study adds evidence on bile acids and NF-κB as targets for liver disease therapy.

## Figures and Tables

**Table 1:** Demographic and laboratory characteristics of the included 4211 patients.

	Non-Cirrhotic CLD (*n* = 985)	Cirrhosis (*n* = 2141)	HCC (*n* = 1085)	*p*
Sex (M/F)	620/365	1338/803	926/159	<0.0001
Age (year)	44.00 (35.00–51.00)	50.00 (43.00–58.00)	52.00 (45.00–59.00)	<0.0001
BMI (kg/m^2^)	24.26 (21.86–26.62)	23.83 (21.50–26.37)	23.88 (21.79–25.95)	0.033
ALT (U/L)	26.00 (17.00–48.00)	30.00 (20.00–55.00)	36.00 (24.00–60.00)	<0.0001
AST (U/L)	25.00 (20.00–38.00)	41.00 (27.00–71.00)	40.00 (28.00–69.75)	<0.0001
GGT (U/L)	25.00 (16.00–54.00)	44.00 (24.00–97.00)	58.00 (33.00–127.00)	<0.0001
ALP (U/L)	77.00 (62.00–99.00)	99.00 (75.00–138.50)	98.00 (76.00–136.00)	<0.0001
ALB (g/L)	40.00 (38.00–43.00)	35.00 (30.00–39.00)	38.00 (35.00–41.00)	<0.0001
PA (mg/L)	198.00 (158.00–231.00)	113.00 (72.75–162.00)	153.00 (110.00–198.00)	<0.0001
TBiL (μmol/L)	11.70 (8.90–16.10)	18.30 (12.05–31.80)	14.60 (10.70–20.08)	<0.0001
DBiL (μmol/L)	4.20 (3.00–5.95)	7.50 (4.50–16.20)	6.20 (4.30–10.20)	<0.0001
CHE (U/L)	7216.00 (5945.50–8329.50)	4525.00 (2925.50–6248.00)	4375.00 (3027.50–6093.50)	<0.0001
GP73 (ng/mL)	46.42 (30.60–66.90)	127.90 (82.56–192.40)	100.50 (60.62–161.50)	<0.0001
PLT (× 10^9^/L)	176.00 (139.00–215.00)	92.00 (57.00–142.00)	134.00 (87.25–181.00)	<0.0001
TBA (μmol/L)	5.00 (3.00–10.00)	21.00 (7.00–53.00)	9.00 (5.00–18.00)	<0.0001
PT (s)	11.00 (10.40–11.70)	12.50 (11.40–14.30)	11.90 (11.10–12.90)	<0.0001

Quantitative variables are expressed as the median (interquartile range). Abbreviation: CLD, chronic liver disease; HCC, hepatocellular carcinoma; F, female; M, male; BMI, body mass index; ALT, alanine transaminase; AST, aspartate transaminase; GGT, gamma glutamyl transpeptidase; ALP, alkaline phosphatase; ALB, albumin; PA, prealbumin; TBil, total bilirubin; DBil, direct bilirubin; CHE, cholinesterase; GP73, Golgi protein 73; PLT, platelet; TBA, total bile acid; PT, prothrombin time. A *p-*value < 0.05 was considered significant. Statistical analysis was performed using SPSS 24.0 software.

## Data Availability

The data presented in this study are available on request from the corresponding author upon reasonable request.

## Supplementary Materials

The following are available online at https://www.mdpi.com/2218-273X/11/2/205/s1, Figure S1: Study flow chart, Figure S2: DCA upregulating the release of GP73 in a dose- and time-dependent manner, Figure S3: DCA upregulating the expression of GP73 in a dose- and time-dependent manner, Figure S4: NF-κB activation upregulates Furin expression, Figure S5: The expression of proteins related to bile acid synthesis and transport in PBS- or DCA-administered mice, Figure S6: Deoxycholic acid (DCA) converts the Golgi into ministacks in Huh-7 cells, Table S1: The primers used for qRT-PCR.
