# Supplementary material for: Deoxycholic Acid Upregulates Serum Golgi Protein 73 through Activating NF-κB Pathway and Destroying Golgi Structure in Liver Disease
Source: Biomolecules. 2021 Feb 2;11(2):205. doi: 10.3390/biom11020205 (PMC7913056; doi:10.3390/biom11020205)
Supplement: Supplementary file 1 [file biomolecules-11-00205-s001.pdf]

Supplementary Figures

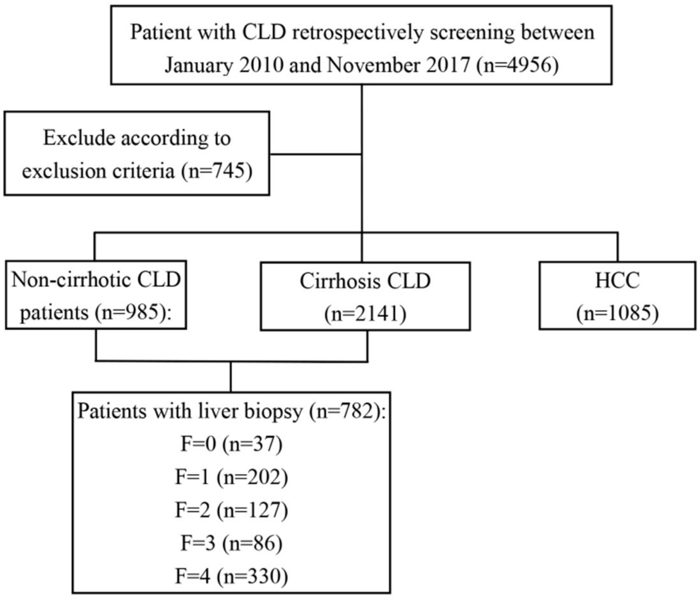

Supplementary Figure S1. Study flow chart

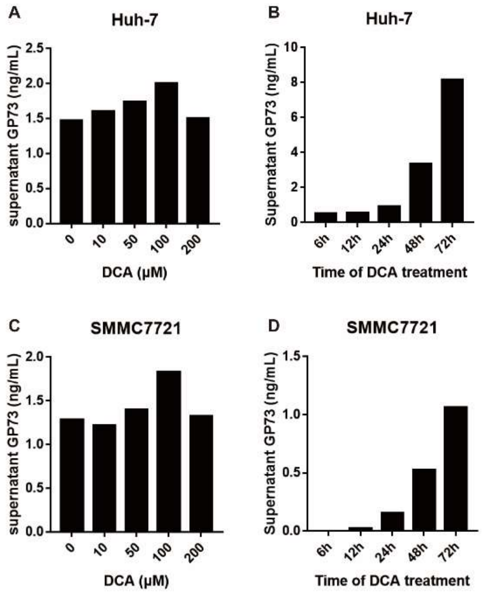

Supplementary Figure S2. DCA up-regulating the release of GP73 in a dose- and time-dependent manner. (A) Huh-7 and (C) SMMC7721 cells were treated with different concentrations of DCA for 72 h. (B) Huh-7 and (D) SMMC7721 cells were treated with 100 μM of DCA with different duration. ELISA was used to detect the GP73 level in cell supernatants.

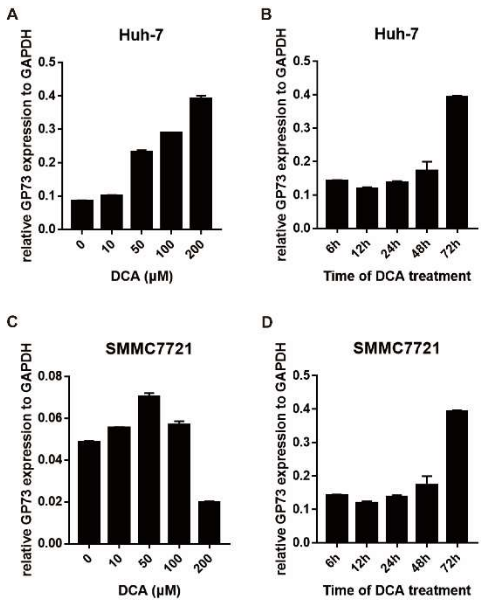

Supplementary Figure S3. DCA up-regulating the expression of GP73 in a dose- and time-dependent manner. (A) Huh-7 and (C) SMMC7721 cells were treated with different concentrations of DCA for 72 h. (B) Huh-7 and (D) SMMC7721 cells were treated with 100 μM of DCA with different duration. qRT-PCR assay was used to detect mRNA level of GP73.

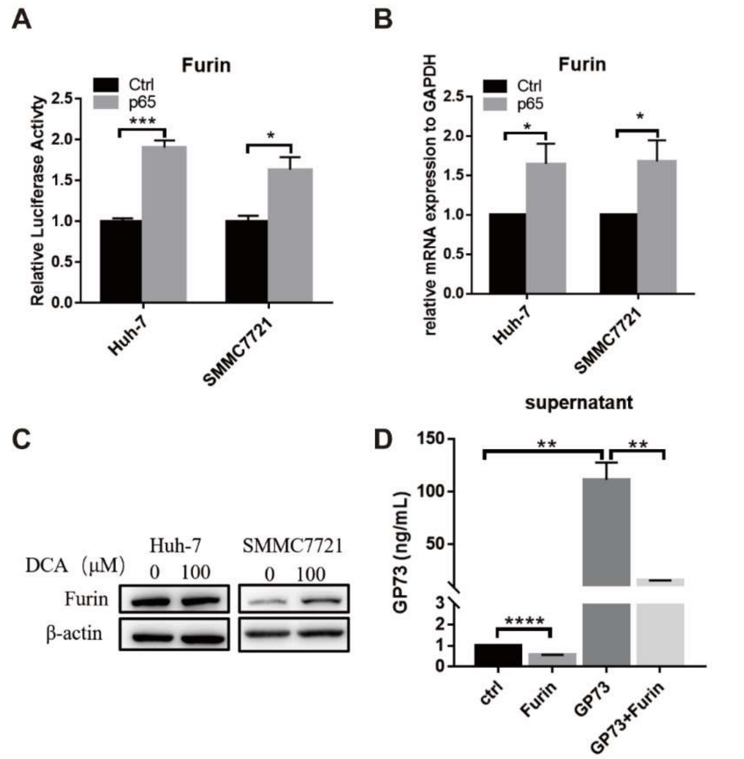

Supplementary Figure S4. **NF-κB activation up-regulates Furin expression.** (A) Huh-7 and SMMC7721 cells were transfected with PGL3 containing promoter of Furin together with control vector PCMV or PCMV-p65. Luciferase activities were measured 48h later. Huh-7 and SMMC7721 cells were transfected with PCMV-p65 or control vector PCMV. qRT-PCR assay of Furin mRNA level (B) and WB assay of Furin were conducted 72 h after transfection. (D) Huh-7 cells were transfected with plasmids as listed. Cell supernatants were collected 48 h later and the levels of GP73 were detected by ELISA.

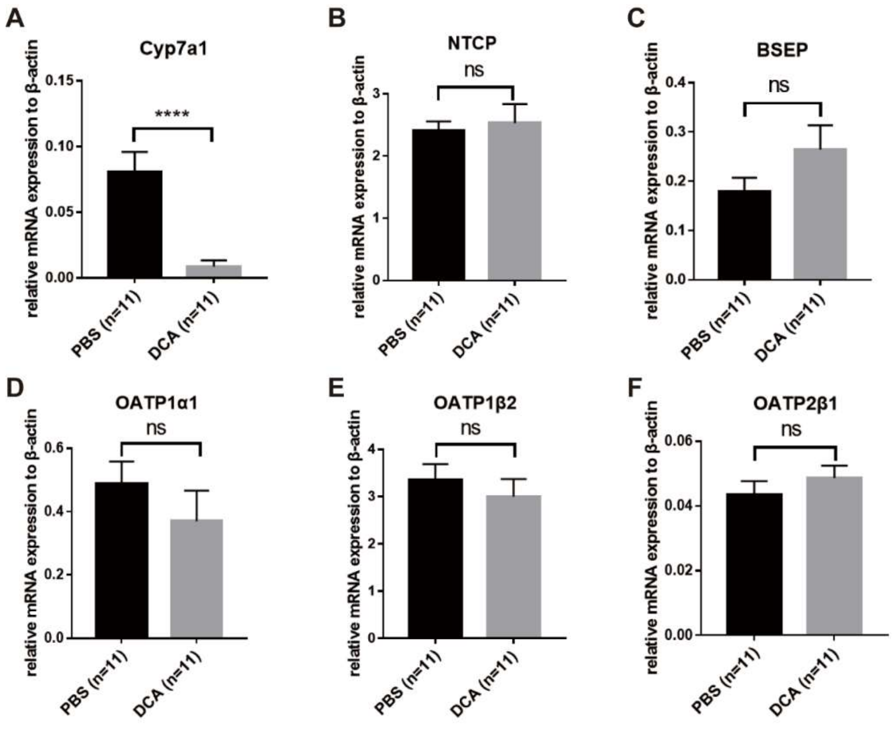

Supplementary Figure S5. The expression of proteins related to bile acid synthesis and transport in PBS or DCA administered mice. qRT-PCR assay of rate-limiting enzyme of bile acid synthesis Cyp7a1 (A) and bile acid transport related protein NTCP (B), BSEP (C), OATP1α1 (D), OATP 1β2 (E) and OATP 2β1 (F).

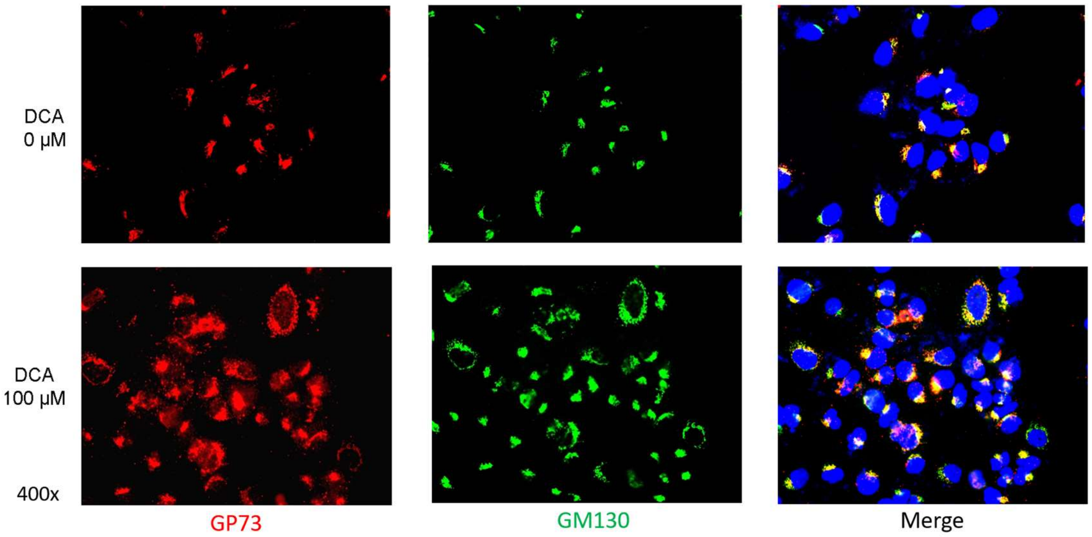

Supplementary Figure S6. Deoxycholic acid (DCA) converts the Golgi into ministacks in Huh-7 cells. Huh-7 cells were treated with 0 and 100 μM DCA for 6 h and co-stained for GP73 (red) and cis-Golgi marker GM130 (green). Nuclei were identified with Hoechst (blue).

Supplementary Table S1: The primers used for qRT-PCR.

The primer sequences used for Real-time RT-PCR

Name	Forward (5′-3′)	Reverse (5′-3′)
hGAPDH	GAAGCAGGCATCTGAGGG	ACCAGGAAATGAGCTTGACA
hGP73	TGGCCTGCATCATCGTCTTG	CCCTGGAACTCGTTCTTCTTCA
hFurin	GCCACATGACTACTCCGCAGAT	TACGAGGGTGAACTTGGTCAGC
mGP73	ATGATGGGATTGGGGAATGGG	TCCAGTAGTTGAAGCCTAGCA
mCyp7a1	GAACCTCCTTTGGACAACGGG	GGAGTTTGTGATGAAGTGGACAT
mNTCP	ATCATGCTCTCGCTTGGCTG	AGGATGGCCAGAGCCTCAAT
mBSEP	TCTGACTCAGTGATTCTTCGCA	CCCATAAACATCAGCCAGTTGT
mOATP1α1	GTGCATACCTAGCCAAATCACT	CCAGGCCCATAACCACACA
mOATP1β2	GCACTGCGATGGATTCAGGAT	AGCTTTGGTCGGTGTAGCTTG
mOATP2β1	TCAGGACTCACATCAGGATGC	CTCTTGAGGTAGCCAGAGATCA
mβ-actin	GGCTGTATTCCCCTCCATCG	CCAGTTGGTAACAATGCCATGT
hβ-actin	CTACAGCTTCACCACCACGG	TCAGGCAGCTCGTAGCTCTTC

The primer sequences used for GP73 and Furin promoter clone

Name	Forward (5′-3′)	Reverse (5′-3′)
GP73-promoter	CCGCTCGAGTGTAGTCCCACCACTCTCGA	CCCAAGCTTCCGGCCTCCGCAGCGGCAAG
Furin-promoter	CCTCGAGGTGAGCATGAGCCTGTTGACAC	CAAGCTTGTGCAACAGTCAGGCTCCTATC

The primer sequences used for mutant GP73 promoter clone

Name	Forward (5′-3′)	Reverse (5′-3′)
GP73-BS1	agagctaaaatgggcaagaaCTATCGCAC AACTC acctgtccccgattatgg	GAGTTGTGCGATAGttcttgcccattttagctctct t agtatagtttacattc

The primer sequences used for chIP-PCR

Name	Forward (5′-3′)	Reverse (5′-3′)
chIP-GP73-BS1	GCTGCCTTAGCACCTAGACA	GCTGACCATACATGACCCCT
chIP-GP73-BS2	TGACAACCGAGGCGAGATGA	AAGCGGCTGTGGGGAAAG
chIP-mGP73	ACTCCAGCAACCACAGCTAATA	CCCCCTCTGCGTGTTAGTACC
